# VP3.15, a dual GSK-3β/PDE7 inhibitor, reduces glioblastoma tumor growth though changes in the tumor microenvironment in a PTEN wild-type context

**DOI:** 10.1016/j.neurot.2025.e00576

**Published:** 2025-03-28

**Authors:** Maria Castello-Pons, Maria A. Ramirez-Gonzalez, Patricia Iglesias-Hernández, Nermina Logo Lendo, Carlos Rodriguez-Martín, Laura Quiralte, Juan-Manuel Sepúlveda-Sánchez, Olaya de Dios, Carmen Gil, Ana Martínez, Pilar Sánchez-Gómez, Sergio Casas-Tinto

**Affiliations:** a Neurooncology Unit, Instituto de Salud Carlos III-UFIEC, Madrid, Spain; b Instituto Cajal-CSIC, Avda. Doctor Arce 37, 28002 Madrid, Spain; c Drosophila Models of Human Disease Unit, Instituto de Salud Carlos III-IIER, Madrid, Spain; d Instituto de Investigaciones Biomédicas I+12, Hospital 12 de Octubre, Madrid, Spain; e Centro de Investigaciones Biológicas Margarita Salas-CSIC, Ramiro de Maeztu 9, 28040 Madrid, Spain; f Centro de Investigación Biomédica en Red en Enfermedades Neurodegenerativas, (CIBERNED), Instituto de Salud Carlos III, Av. Monforte de Lemos, 3-5, 28029 Madrid, Spain; g PhD Programme on Biomedical Sciences and Public Health, Universidad Nacional de Educación a Distancia, UNED-ISCIII 28040 Madrid, Spain

**Keywords:** GSK-3β, PDE7A, PTEN, GAL9, Tumor microenvironment, Macrophages

## Abstract

Glioblastoma (GB) is an incurable cancer of the brain, and there is an urgent need to identify effective treatments. This may be achieved by either identifying new molecules or through drug repurposing. To ascertain the therapeutic potential of known GSK-3β and/or PDE7 inhibitors in GB, a drug screening was conducted using a *Drosophila melanogaster* glioma model. VP3.15, a dual inhibitor with anti-inflammatory and neuroprotective roles in multiple sclerosis, was selected for further investigation. VP3.15 demonstrated robust anti-tumor efficacy against a panel of human and mouse GB cells; however, its capacity to inhibit orthotopic growth was only observed in a wild-type PTEN cell line. The *in vivo* dependence on PTEN was further suggested with the results in fly gliomas. The analysis of the VP3.15-treated tissues revealed a notable reduction in the number of myeloid cells and in the degree of vascularization. Mechanistic studies indicate that VP3.15 diminishes the production of GAL9, a key molecule that stimulates pro-angiogenic macrophages. Our findings substantiate the pro-tumoral function of GSK-3β, which might depend on the *PTEN* genetic status. Furthermore, we have delineated the therapeutic potential of VP3.15, which acts through the inhibition of the supportive role of the GB microenvironment. This molecule could be safely and effectively utilized after PTEN characterization in GB patients.

## Introduction

Glioblastoma (GB), classified as a grade 4 astrocytoma, is one of the most aggressive forms of cancer, as well as the most frequent malignant primary brain tumor [1]. The standard treatment for GB is surgical removal of the tumor followed by radiotherapy and chemotherapy with temozolomide (TMZ). However, the invasiveness and proliferation rate of tumor cells, as well as their high resistance to conventional therapies favor the recurrence of GB, ultimately resulting in the death of the patients within 15–20 months after the initial diagnosis [1]. The development of novel pharmacological agents, especially those capable of reaching the brain, is necessary to enhance the survival of these patients, who have not seen a therapeutic improvement in the last decades.

The aggressiveness of glioma cells is sustained by their close relationship with the surrounding stroma, particularly with the vascular and immune cells, the latter of which constitute up to 50 ​% of the tumor mass content in some cases of GB. Moreover, the immune compartment of GB is enriched with myeloid cells that possess strong immunosuppressive and pro-angiogenic properties [2]. Several groups have proposed that effectively eradicating glioma cells requires the targeting of these pro-tumoral myeloid cells as well. However, thus far none of the proposed molecules has achieved clinical success [3]. In a similar manner, anti-angiogenic strategies have been unsuccessful in prolonging the overall survival of patients with GB to date [4].

Notwithstanding the above, there is a consensus that genes regulating the interaction between glioma cells and the surrounding microenvironment remain important as therapeutic targets. That is exemplified by *PDE7B* [5] and *GSK-3β* [6]. Indeed, overexpression of PDE7B has been observed in GB, resulting in enhanced tumor growth and aggressiveness in murine models [5]. Furthermore, the elevation of intracellular cAMP levels through the inhibition of cAMP phosphodiesterases with various PDE2/4/7 inhibitors may prove to be a beneficial therapeutic strategy, leading to the suppression of glioma cells [7,8]. Likewise, emerging evidence suggests that GSK-3β functions as a tumor promoter in glioma, playing a key role in proliferation, resistance to radio-chemotherapy, and activation of invasion [9,10]. Moreover, the inhibition of GSK-3β with small molecules has been demonstrated to enhance the effect of TMZ in glioma cells and to reduce GB growth *in vivo* [11,12].

Our research group has extensive experience in the design and development of GSK-3β and PDE7 inhibitors, primarily for CNS diseases [[13], [14], [15]]. This includes the development of the first dual GSK-3β/PDE7 inhibitors described to date [16]. In this study, we screened a diverse range of chemically distinct small molecule inhibitors of GSK-3β, PDE7 and/or dual GSK-3β/PDE7 in a *Drosophila melanogaster* glioma model with the objective of identifying the most promising candidates for future translational studies. We selected VP3.15, an iminothiadiazol dual inhibitor of the two targets, based on its superior anti-tumor capacity. *In vitro* viability tests using different GB models confirmed its potential for chemotherapeutic treatment. However, in orthotopic xenografts and allografts, the effect of VP3.15 was limited to the inhibition of the growth of a *PTEN* wild-type GB, accompanied by a reduction in tumor vascularity. This PTEN dependency was subsequently validated in *Drosophila melanogaster* models of glioma. The results of the mechanistic studies indicate that the effect of VP3.15 in the presence of PTEN is mediated through a reduction in Gal9 expression and the consequent inhibition of the recruitment of pro-tumoral macrophages. In conclusion, the presented results indicate that VP3.15 may serve as a highly efficacious inhibitor of PTENwt glioma growth, influencing the interaction between tumor cells and their supportive microenvironment.

## Materials and methods

### Drug synthesis and purification

All the compounds were synthetized, isolated, purified and characterized in CIB–CSIC laboratories following previously described procedures. Table S1a shows the chemical structure, the IC_50_ values against their targets and the reference for their synthetic procedure.

### In vivo assays

#### Drosophila melanogaster UAS/GAL4 expression binary system

We used the binary expression system Gal4/UAS to modulate gene expression. Briefly, the *Gal4* transcriptional activator from *Saccharomyces cerevisiae* was expressed under the control of a specific promoter active in specific tissues or cells. We used *repo*, a regulatory sequence that controls the expression of the gene *reverse polarity* and is active specifically in glial cells [17]. Next, the activating sequence *UAS (Upstream Activating Sequence)* was fused to a gene of interest. By crossing two parental lines, one carrying the gene of interest fused to the UAS sequence, and the other one containing the gene encoding GAL4 under the control of a tissue-specific promoter, the offspring activates the expression of the gene or dsRNA of interest in a specific tissue [18].

#### *Drosophila* stocks

Fly stocks were maintained at 25 ​°C, 60 ​% humidity and 12/12 ​h light/dark cycles. Fly stocks used were *UAS-LacZ* (BL8529), *UAS-myrRFP* (BL7119), *repo-Gal4* (BL7415), *dnc-GFP* MiMIC (BL43732), *UAS-GSK3*^*DN*^ (BL5359)*, UAS-dnc RNAi* inserted in chromosome III (BL27250) and *UAS-PTEN RNAi* (BL25841) from the Bloomington Stock Center (https://flystocks.bio.indiana.edu/). The stocks *UAS-dnc RNAi* inserted in chromosome II (VDRC 107967) and *UAS-GSK3 RNAi* (VDRC 101538) from VDRC (https://shop.vbc.ac.at/vdrc_store/), and *UAS-dp110CAAX and UAS-EGFR* gifted by R. Read [19].

GB model was generated as previously described [19]. Briefly, we used the specific pan-glial promoter *repo* to express the constitutively active forms of *EGFR* and *PI3K* in glial cells and precursors, under the control of the UAS/Gal4 system. This genetic combination has been proved to reproduce GB features in *Drosophila* brain and is a well stablished model [[19], [20], [21]]. In addition, we expressed a myristoylated form of the *red fluorescent protein* (UAS-myrRFP) under the control of *repo-Gal4* to visualize the membrane of glial/GB cells.

#### Drug screening

The drugs were administered to *Drosophila* larvae through the diet mixed with the food. The drugs were diluted in dimethyl sulfoxide (DMSO) to a final concentration of 50 ​mM–200 ​mM according to their solubility:

VP3.15 and VP1.14 were dissolved at 100 ​mM, VP1.15, S14, TC3.6, TDZD8, Tideglusib and VP2.51 ​at 200 ​mM and VP0.7 was dissolved at 50 ​mM due to challenges in achieving complete dissolution. Even if VP07 was not fully dissolved in DMSO, we decided to incorporate it into the experiments.

The diluted compounds were mixed into standard food heated at 56 ​°C, a temperature at which it remains in liquid form, enabling the mixture of the compound with the food.

We prepared 5 ​mL of food mixed with 5, 20, or 35 ​μl of each compound. The highest DMSO concentration was found to be toxic to the larvae so 5 and 20 ​μl amounts were tested.

#### Drosophila Larval brain dissection and immunostaining

We dissected the larvae in phosphate buffered saline (PBS). We held the head of the larva with tweezers, and we inserted forceps through the opening of the section. Next, we pushed until the internal wall and viscera were exposed to the exterior. Finally, the viscera of the larva were removed, and the brain was located and separated from the rest of the body.

We fixed *Drosophila* tissues with 4 ​% formaldehyde (PFA) for 20 ​min at room temperature (RT). Next, we washed the samples three times in PBS 1x + 0.3 ​% Triton X-100 (PBT) for 10 ​min under stirring at RT. Next, we blocked the samples in (0.3 ​% PBT ​+ ​5 ​% BSA) under agitation for 30 ​min at RT. We incubated the primary antibody in blocking solution at 4 ​°C overnight. Next, we washed the samples in PBT (3 ​× ​15 ​min) and we incubated the secondary antibody (fluorochrome-conjugated) in blocking solution for 2 ​h in the dark and under constant agitation at RT. Finally, we washed the samples in PBT (3 ​× ​15 ​min) and we mounted the brains on microscope slides, using mounting medium (Vectashield with DAPI), and covering the slide with a coverslip until analysis.

We used the primary antibody anti-Repo (DSHB, 1/100), which recognizes the transcription factor encoded by the *repo* gene, to mark the nuclei of glial cells.

Secondary antibodies (Thermofisher): anti-mouse Alexa 488. DNA was stained with 2-(4-amidinophenyl)-1H-indole-6-carboxami-Dine (DAPI) at 1 ​μM in Vectashield mounting media (Vector Laboratories).

#### Drosophila GB quantification and analysis

*Drosophila* brain samples were mounted in Vectashield with DAPI and we acquired confocal images using a 20x or 40× oil objectives, 1024 ​× ​1024 resolution, and images were taken every micrometer in Z sections. We visualized the number of normal glia (controls) or transformed glia (GB samples) cells with the anti-repo staining. To quantify GB progression, we counted the number of repo positive cells per brain hemisphere using IMARIS (Bitplane) software. Each point represents the number of repo-positive cells in one hemisphere of the brain.

#### GB cells

We used 4 mouse and two human GB cell lines (Fig. 3a). The SVZ-wt and SVZ-vIII cell lines were previously characterized in our laboratory [22]. They were derived from p16/p19 ko subventricular zone (SVZ) progenitors after the overexpression of EGFRwt or the deleted isoform EGFRvIII. Even though they were generated from C57/BL6 mouse tissue they need to be injected into immunodeficient mice to generate tumors. GL261 cells were acquired commercially and NPE-IE cells were kindly donated by Stephen Pollard [23]. These two cell lines form tumors when injected into immunocompetent C57/BL6 mice. 12O89 ​cells were derived from a patient's sample after subject's written consent (according to the Declaration of Helsinki) and with the approval of the Ethical Committee at Hospital 12 de Octubre (Madrid, Spain) (CEI-18/024). RG1 cells were kindly donated by Dr. Rosella Galli [24]. Both human cells form tumors when injected into the brains of Nude mice. All cells express the Luciferase gene as a reporter to monitor tumor growth *in vivo*.

All the cells were maintained in Complete Medium (DMEM/F12 (Thermo Fisher Scientific) supplemented with B27 (1:50) (Thermo Fisher Scientific); penicillin-streptomycin (1:100) (Lonza); 0.4 ​% heparin (Sigma-Aldrich); and 40 ​ng/ml EGF and 20 ​ng/ml bFGF2 (Peprotech). They were maintained at 37 ​°C/5 ​% O2 and passaged after enzymatic dissociation using Accumax (Millipore).

#### Orthotopic mouse glioma models

Animal experiments were reviewed and approved by the Research Ethics and Animal Welfare Committee at our institution (Instituto de Salud Carlos III, Madrid) (PROEX 055/19), in agreement with the European Union and national directives. The tumors were established after the injection of 50,000–300,000 ​cells (depending on the cell line) in 2 ​μl of complete medium with a Hamilton syringe into the brains of C57/BL6 mice (for NPE-IE and GL261 ​cells) or Nude mice (for the rest). The injections were made into the following coordinates: A–P, −0.5 ​mm; M–L, +2 ​mm, D–V, −3 ​mm (related to Bregma) using a Leica Stereotaxic device. Three days after the tumor implantation, animals started receiving treatment. For that, the stock of VP3.15 (100 ​mg/ml in DMSO) was further dissolved in 5 ​% Tocrisolve (Tocris) in saline and injected intraperitoneally (i.p.) (10 ​mg/kg/day, 5 days/week). Control animals were treated with 5 ​% Tocrisolve in saline. For intravenous treatment, the stock of VP3.15 was dissolved in saline and injected 2 days/week (5 ​mg/kg). For subcutaneous treatment the stock of VP3.15 was dissolved with 5 ​% Trocrisolve in saline and injected 5 days/week (30 ​mg/kg). TMZ (MedChemExpress) was dissolved in PBS 1 ​% BSA and injected i.p. (5 ​mg/kg/day, 5 days/week). Tumor growth was monitored by bioluminiscence in an IVIS equipment (PerkinElmer) after intraperitoneal injection of D-luciferin (75 ​mg/kg; Thermo Fisher Scientific). Animals were sacrificed when they showed signs of disease, the brains were dissected out and processed for cellular and molecular analysis.

### In vitro treatment

For viability testing, 5000 ​cells were seeded in triplicate in 96-well plates. After 24 ​h, cells were treated with VP3.15 (at the indicated concentrations) and viability was measured after 72 ​h of treatment using Alamar Blue (Thermo Fisher Scientific). For that, the Alamar Blue reagent was added to the cell media (1:10) and Absorbance at 570 ​nm (using 600 ​nm as a reference wavelength) was measured in a TECAN equipment (Life Sciences). For all cell lines, the absorbance of each well was related to that of the controls, which were treated with DMSO (1:1000).

#### Inmunohistochemical (IHC) staining and quantification

Mouse brains were fixed in 4 ​% PFA, embedded in paraffin and sectioned (5 ​μm) in a microtome (Leica). For IHC staining with different antibodies (Table S1b), the slides were preheated for 30 ​min at 65 ​°C, and the BOND RXm automated advanced staining system (Leica Biosystems) programmed with the specific protocols (Table S1c). All the stained slides were scanned using the NanoZoomer-SQ microscope slide digitizer (Hamamatsu). QuPath software was used for the quantification, setting two different methods depending on the specific antibody: positive-cell detection (for Ki67 staining) and pixel classification (for Endomucin, CD68 and CD206 staining). The first one measures the percentage of positive cells with respect to negative cells in a specific area. In the second one an intensity threshold was established in the regions of interest for each specific marker. Signal above that threshold was considered positive and the percentage of positive staining related to the total area was calculated. Table S1d provides detailed information on the parameters and settings employed in all quantifications.

#### Quantitative reverse-transcriptase PCR (qRT-PCR)

Frozen samples of the tumor tissue were used for RNA extraction, which was performed using the RNA isolation Kit (Roche). Equivalent amounts (1 ​μg) of purified RNA were reverse transcribed using PrimeScript RT Reagent Kit (Takara) and quantitative real time PCR (qRT-PCR) reactions were performed using the Light Cycler 480 (Roche Diagnostics) with the SYBR Premix Ex Taq (Takara) and specific primers for each gene (Table S1b). Relative gene expression was calculated using the double delta Ct method.

#### Western Blot (WB) analysis

Protein extracts from cell pellets or glioma tissue samples were prepared by resuspending them in lysis buffer (50 ​mM Tris (pH 7.5), 300 ​mM NaCl, 0.5 ​% SDS, and 1 ​% Triton X-100), followed by 15 ​min (100 ​°C) incubation. The lysed extracts were centrifuged at 13,000 ​g for 10 ​min (RT) and the protein content was determined using the BCA Protein Assay Kit (Thermo Fisher Scientific). 30 ​μg of protein were resolved by 12 ​% SDS-PAGE and then transferred to a nitrocellulose membrane (Hybond-ECL, Amersham Biosciences). The membranes were blocked and then incubated with the primary (1 ​h at RT) and the respective secondary antibody (2 ​h at RT) (Table S1b) diluted in TBS-T (10 ​mM Tris-HCl (pH 7.5), 100 ​mM NaCl, and 0.1 ​% Tween-20). Proteins were visualized by enhanced chemiluminescence with ECL (Pierce) using the Imager 800 (Amersham Biosciences) and the signal was quantified by ImageQuant TL software (Cytiva).

### In silico analyses

The generation of the single-cell transcriptomic (scRNAseq) integrated atlas has been previously described [25]. In summary, we downloaded different scRNAseq databases available in public repositories of normal brain [26], lower grade gliomas (LGG), and GB both newly diagnosed and recurrent [27]. Subsequent data processing and analysis were performed using Seurat package v4.3.0. Batch-corrected dimensionality reduction was performed using Harmony [28], correcting for sample type. Cell types were manually identified based on the canonical markers described in previous studies and confirmed by scCATCH (single cell Cluster-based Annotation Toolkit for Cellular Heterogeneity) [29].

To analyze and visualize the correlation between *GSK3b* and *PDE7A/B* expression, we used the GlioVis data portal (http://gliovis.bioinfo.cnio.es) and the TCGA (The Cancer Genome Atlas) and the CGGA (Chinese Glioma Genome Atlas) cohorts (including GB and LGG).

### Statistical analysis

Data presentation and statistical analysis was performed using GraphPad Prism 5 software. Differences between pairs of experimental groups were analyzed by two-tailed un-paired Student's *t*-test and one-way ANOVA. To assess survival differences in mouse experiments we used the Kaplan–Meier method, evaluated with a two-sided log-rank test. P values ​< ​0.05 were considered significant (∗p ​< ​0.05; ∗∗p ​< ​0.01; ∗∗∗p ​< ​0.001; ∗∗∗∗p ​< ​0.0001; n.s., non-significant). All quantitative data presented are the mean ​± ​SEM.

## Results

### PDE7 and GSK-3β expression in GB patients

**Fig. 1:**
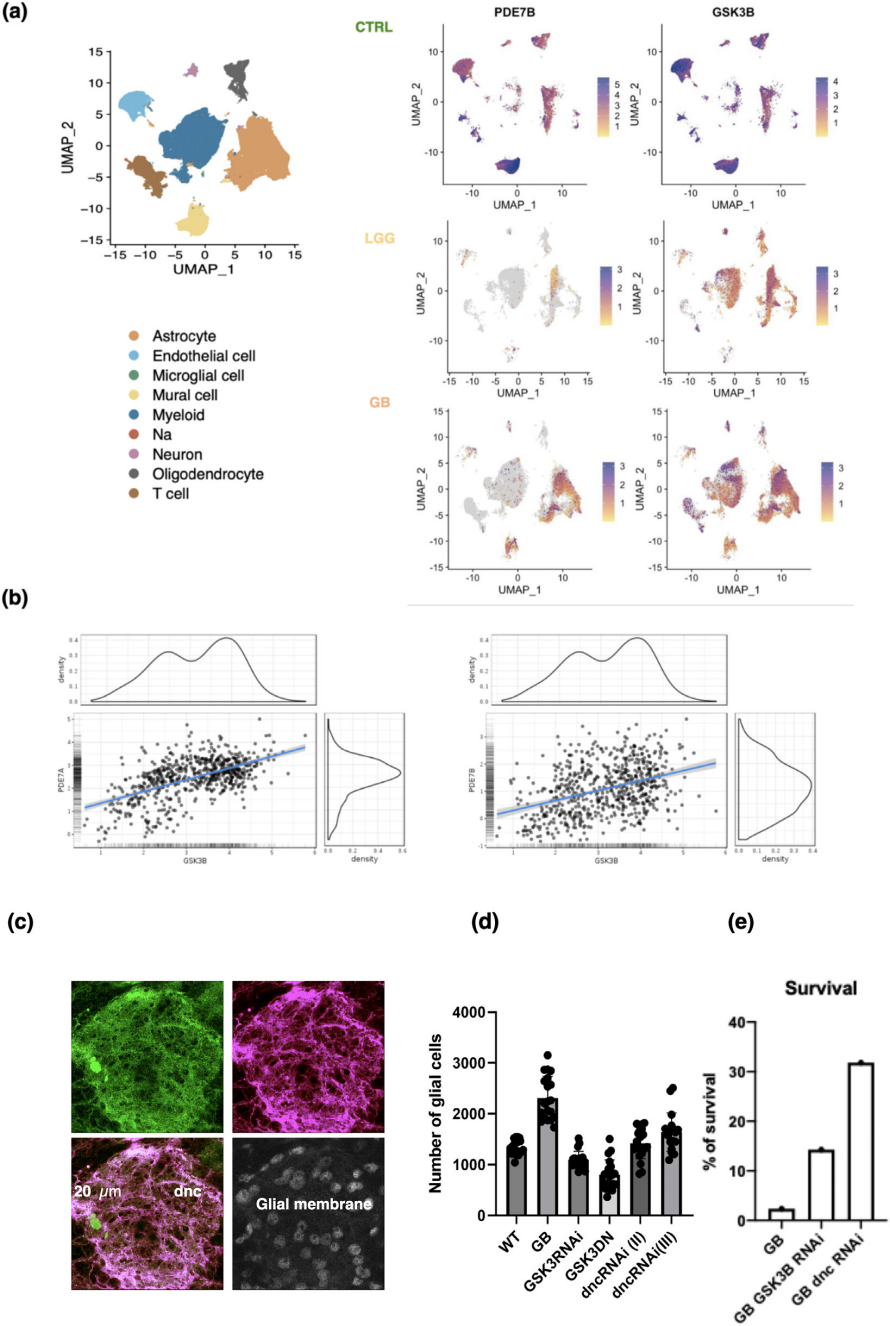
**Analysis of *GSK3B* and *PDE7* expression in GB. a)** UMAP plot annotated according to each cell type of the integrated object (control and glioma scRNAseq databases). **b)** UMAP plot of cells with positive *PDE7B* and *GSK3B* expression in normal brain (CTRL), lower grade gliomas (LGG) and glioblastomas (GBM). **c)** Data from GlioVis database (http://gliovis.bioinfo.cnio.es/) showing the correlation of *GSK3B* expression with *PDE7A* or *PDE7B* in the CCGA (Chinese Glioma Genome Atlas) cohort of gliomas. **c)** Amplification of confocal images showing brain tissue from *Drosophila melanogaster* bearing a genetically induced GB. Dunce (dnc) is the PDE orthologue in *Drosophila* (shown in green) is upregulated in GB tissue (GB membrane in red, and GB cells nuclei in gray). **d)** Graphic showing the number of GB cells in *Drosophila* upon *GSK3* or *dnc* knockdown (RNAi) or dominant negative expression GSK3^DN^. **e)** Survival of flies reaching adulthood upon GB induction, GB and *GSK3* or *dnc* knockdown by RNAi.

We conducted an *in silico* analysis integrating publicly available data from scRNAseq studies of non-tumoral brain and gliomas [26,27] with a focus on the analysis of *PDE7* and *GSK-3β* expression in gliomas. The analysis revealed the presence of distinct immune (macrophages, microglia and T cells), astrocytic and vascular (endothelial cells and pericytes) cell groups (Fig. 1a-left). The analysis of *PDE7B* and *GSK-3β* transcription showed that both genes were expressed in various cell types within the normal brain. However, a notable increase in *PDE7B* and *GSK-3β* expression was observed in GB samples compared to LGG (Fig. 1a-right). In GB, *PDE7B* transcription is predominantly observed in astrocytic cells (including neoplastic cells) and pericytes, whereas *GSK-3β* is expressed in a ubiquitous manner (Fig. 1a-right). Subsequently, we examined the correlation between *GSK-3 β* and *PDE7A* or *PDE7B* expression levels in the GlioVis database. The results demonstrate a robust positive correlation (Fig. 1b) (p-value ​= ​0.00 in both cases), suggesting that the elevation of *GSK-3β* and *PDE7* occurs concurrently.

### Relevance of GSK-3 and PDE7 in the Drosophila GB model

To ascertain the role of GSK-3 and PDE7 in the progression of glial tumors *in vivo*, a GB model in *Drosophila melanogaster* [19,21] was employed. The expression of *dunce (dnc)*, the orthologue of *PDE7* in flies, was determined in *Drosophila* larvae bearing a GB. We used a green fluorescent protein reporter (*dnc-GFP*) to monitor the activity of *dnc* promoter. Confocal images of the brain lobes showed an increase in the GFP signal in GB samples suggesting that *dnc* expression is upregulated in GB brains in *Drosophila* (Fig. 1c).

Next, to ascertain the functional contribution of *GSK-3* and *dnc* to GB progression, the number of glial cells per brain lobe was counted in WT, GB flies, and GB flies upon *GSK3* or *dnc* knockdown (RNAi) or the expression of *GSK-3* dominant negative (DN) forms. The results show that the interference with *GSK-3* and *dnc* expression or function markedly reduces the number of cells in the GB (Fig. 1d). Subsequently, we examined the lethality resulting from the induction of a GB in larvae. The results show that the lethality induced by the induction of a GB in fly larvae can be rescued by the knockdown of *GSK-3* or *dnc* in GB cells (Fig. 1e). These findings suggest that *GSK-3* and *dnc* are necessary for GB progression *in vivo*, making them promising candidates for therapeutic intervention in these tumors.

### Drug screening in the Drosophila GB model

**Fig. 2:**
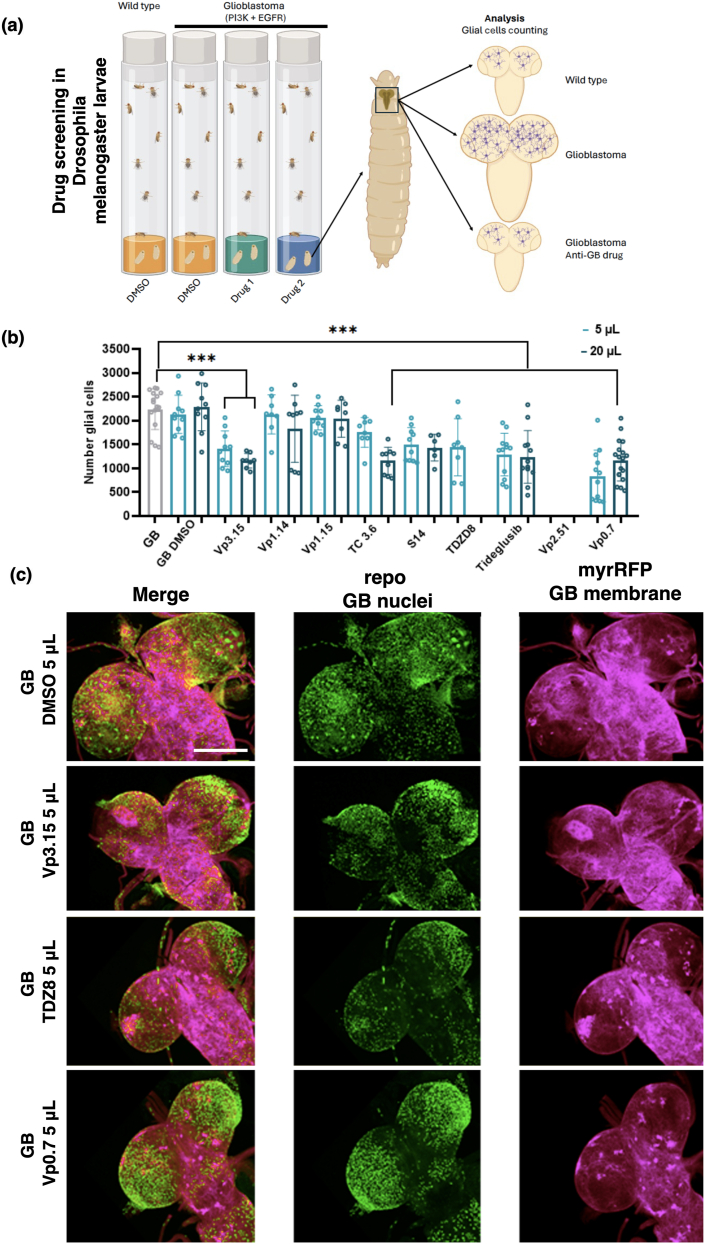
**Drug screening in Drosophila larvae. a)** Schematic representation of drug screening performed in *Drosophila melanogaster* larvae. Drugs were mixed in the food using 5, 20 or 35 ​μl per 5 ​ml. **b)** Graph showing the number of GB cells per brain lobe treated with vehicle (dimethyl sulfoxide, DMSO) of drugs. **c)** Representative images of *Drosophila* larvae brains with a genetically induced GB treated with 5 ​μl of DMSO, VP3.15, TDZ8 or VP0.7. Glial cells are marked in green (repo); glial membrane is marked in red (myr-RFP) and nuclei of all cells is marked in blue (DAPI).Scale bar: 100 ​μM.

Next, anti-GB effect of a number of chemically diverse GSK-3 and PDE7 inhibitors was evaluated. These compounds had been previously designed and synthetized in our laboratory as pharmacological tools to modulate specifically these two targets (Table S1a). Therefore, a selection of four GSK-3, three PDE7 inhibitors, and three multitarget compounds [14], capable of inhibiting both GSK3 and PDE7 (Table S1a), was chosen for further analysis. The anti-GB effects of these ten drugs were evaluated at two distinct concentrations. A total of 5 ​μl and 20 ​μl of each compound stock, including a control containing only DMSO, were added to 5 ​ml of fly food. The viability of the flies was assessed, and the number of glial cells was counted. The genetically-induced GB co-expresses the gene for *red fluorescent protein* (*UAS-myrRFP*). This enables the selection of brains containing glial tumors (red fluorescence positive) and the enumeration of glial cells stained with the specific antibody repo (Fig. 2a). The compounds (Table S1a) VP3.15, TC 3.6, S14, TDZD8, Tideglusib and VP 0.7 significantly reduced the number of glial cells in GB. The administration of high concentrations of TDZD8 (20 ​μl) and VP 2.51 (5 ​μl and 20 ​μl) resulted in lethal outcomes. Conversely the addition of VP 1.14, VP 1.15 and TC 3.6 did not elicit a notable impact on the number of glial cells within the brains of the flies (Fig. 2b and c).

The findings suggest that our GSK3 inhibitors may prove more efficacious than the PDE7 inhibitors in preventing the progression of GB in *Drosophila*. Moreover, dual inhibition of these two targets by compound VP3.15 resulted in a significant impairment of GB growth at both concentrations tested. This drug was selected for further investigation of its potential to inhibit GB progression.

### Effect of VP3.15 in the in vitro and in vivo growth of mouse and human GB cells

**Fig. 3:**
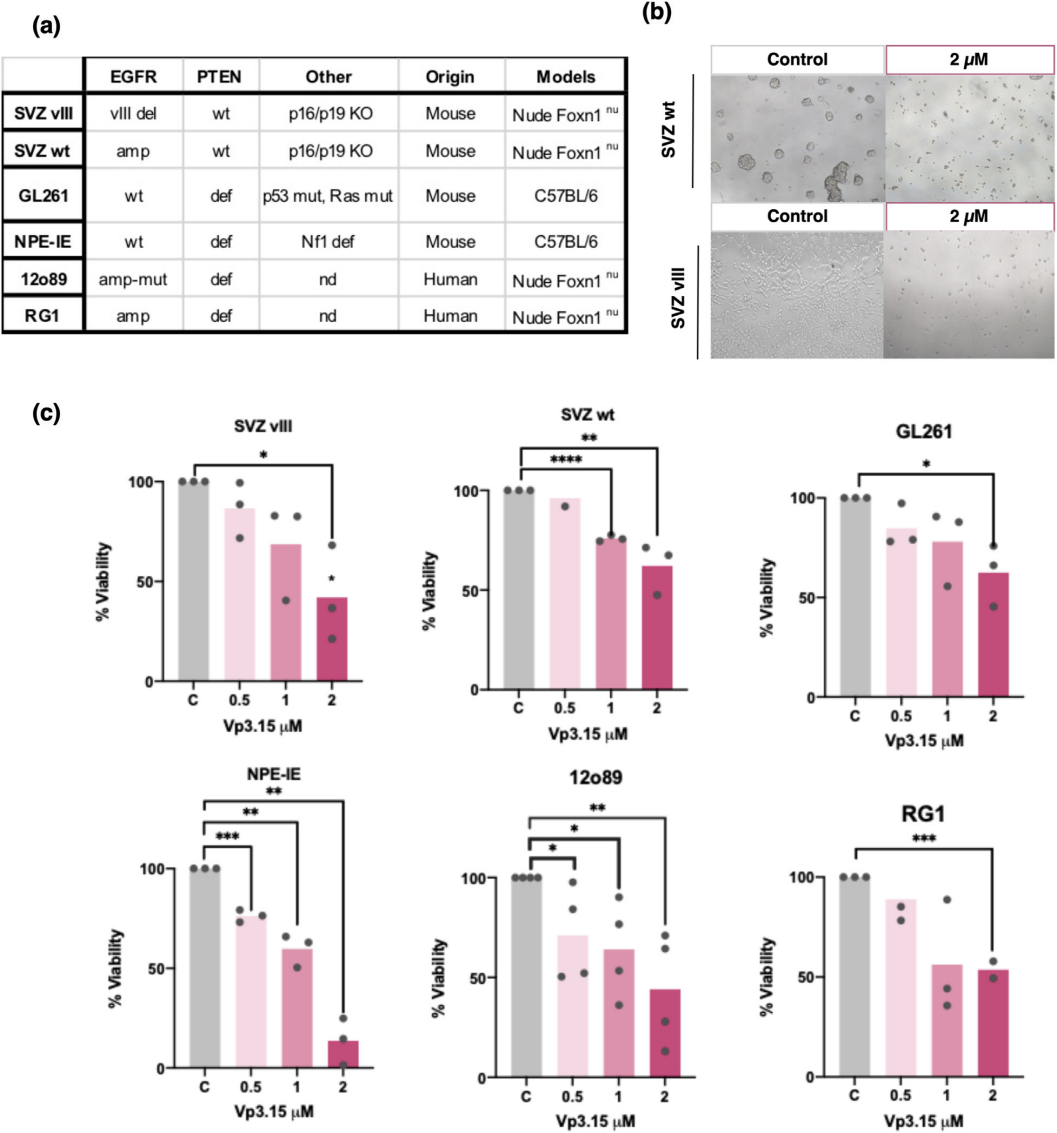
***In vitro* response of GB cells to Vp3.15. a)** The table displays the list of GB cells used in this study, along with their known genetic features, their origin (human or mouse tumors), and the mice that are used to grow them *in vivo* after orthotopic implantation (Models). **b)** Representative contrast-phase images of two distinct GB cell lines treated either with dimethyl sulfoxide (DMSO) (control) or VP3.15 ​at 2 ​μM over a 72-h period. **c)** The various cell lines were grown in the presence of DMSO (1:1000) or increasing concentrations of VP3.15. Following a 72-h incubation period, the viability of the cells was determined by measuring the absorbance after incubation with Alamar Blue. The absorbance measured in the presence of DMSO was designated as 100 ​%. The experiment was repeated 3 times. ∗P ​≤ ​0.05; ∗∗P ​≤ ​0.01; ∗∗∗P ​≤ ​0.001; ∗∗∗∗P ​≤ ​0.0001. del: deletion, amp: amplification, wt: wild-type, mut: mutant, def: deficient, KO: knock out, nd: not determined.

To further examine the anti-tumor capacity of VP3.15, we initially conducted a viability assay on a panel of primary GB cells derived from human or mouse GB tumors with diverse genetic characteristics (Fig. 3a). The cell cultures were incubated for three days in the presence of increasing concentrations of the compound and the viability was measured with Alamar Blue . While there were some differences in the sensitivity of each individual cell line, a significant decrease in the viability was observed in all of them in the presence of 2 ​μM of VP3.15 compared to the control (cells treated with the vehicle DMSO) (Fig. 3b and c).

**Fig. 4:**
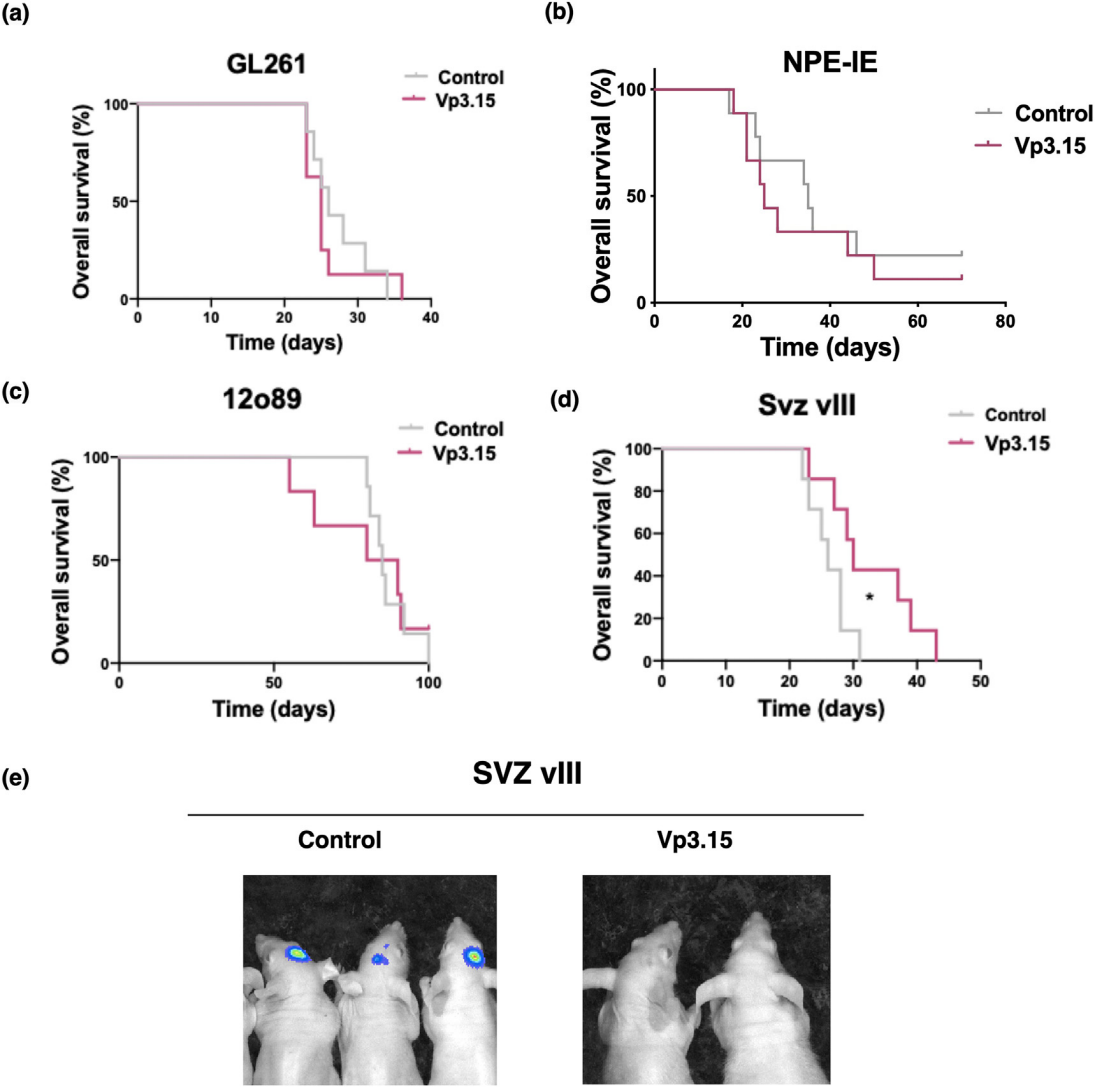
***In vivo* analysis of the effect of VP3.15 in GB. a-d)** Kaplan-Meier overall survival curves of mice that were orthotopically implanted with GL261 **(a)**, NPE-IE **(b)**, 12O89 **(c)** or SVZ-vIII **(d)** cells. The animals were administered i.p. injections of the vehicle (control) or VP3.15 (10 ​mg/kg/day, 5 days/week), and they were sacrificed when they exhibited symptoms of the disease (n ​= ​7). **(e)** Representative image of the bioluminescence analysis of animals two weeks after the injection of SVZ-vIII cells, which express the luciferase reporter. ∗P ​≤ ​0.05.

**Fig S1:**
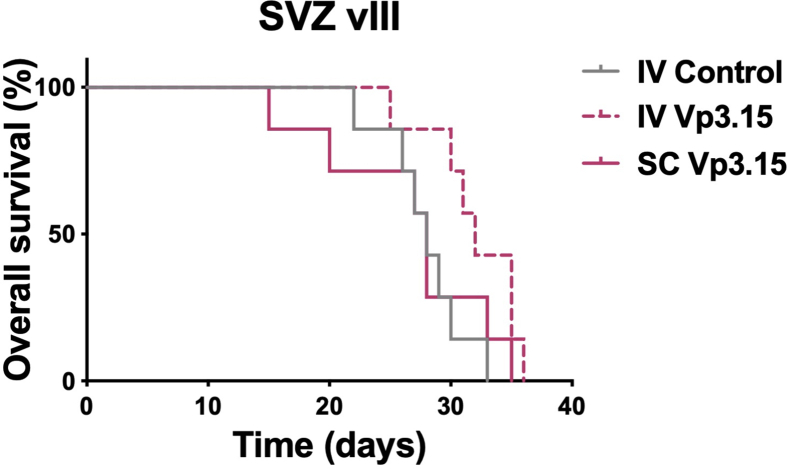
Test of different administration routes for VP3.15. Kaplan-Meier overall survival curves of mice that were orthotopically implanted with SVZ-vIII cells and treated with intravenous (IV) injections of vehicle or VP3.15 (5 ​mg/kg/day, 2 days/week), or subcutaneous injections of VP3.15 (30 ​mg/kg/day, 5 days/week) (n ​= ​7).

**Fig S2:**
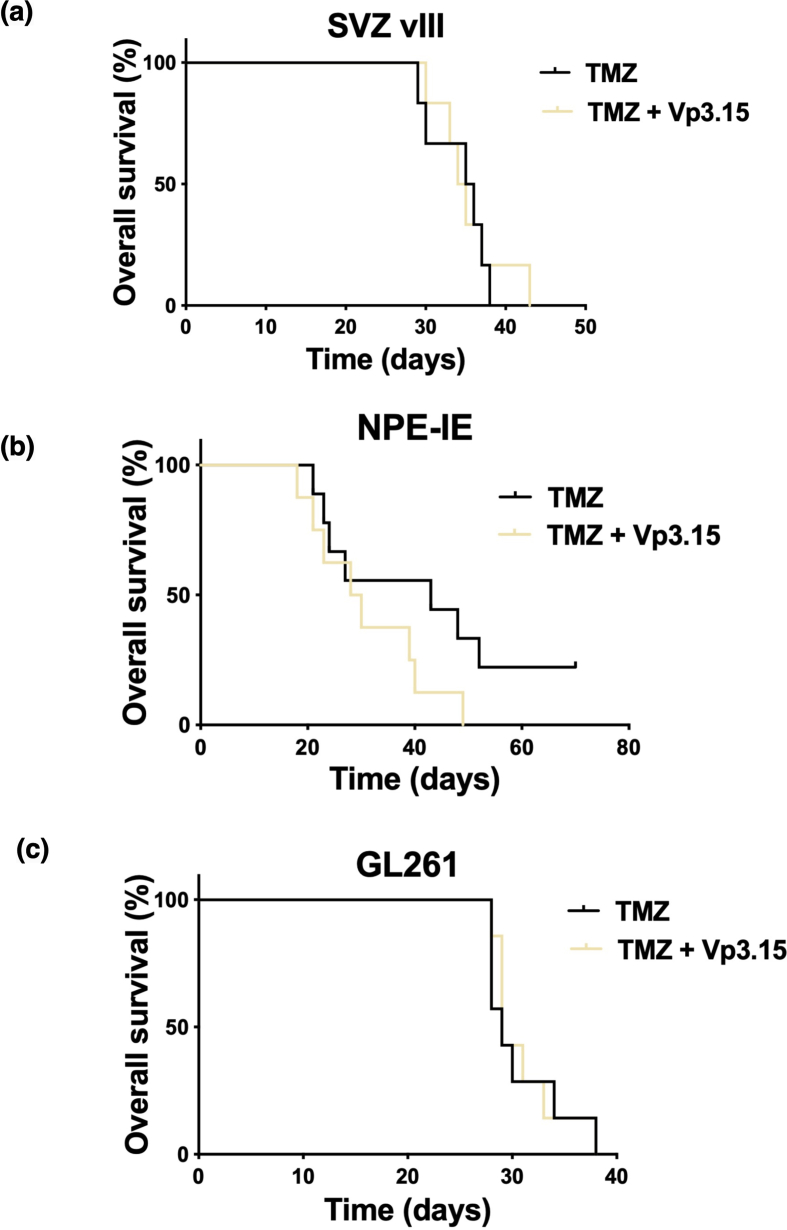
Combinatorial treatment of Vp3.15 and TMZ in different *in vivo* GB models. Kaplan-Meier overall survival curves of mice that were orthotopically injected with SVZ-vIII **(a),** NPE-IE**(b)** and GL261**(c)** cells and treated with i.p. injections of TMZ (5 ​mg/kg/day, 5 days/week), alone or in combination with i.p. injections of VP3.15 (10 ​mg/kg/day, 5 days/week) (n ​= ​7).

Next, to validate these results *in vivo*, we injected the GB cell lines into the brain of mice that were then treated systemically with 10 ​mg/kg of VP3.15 (i.p. injections). We analyzed the overall survival of the animals, and the results show that the drug did not inhibit the growth of GL261 (Fig. 4a), NPE-IE (Fig. 4b) or 12O89 (Fig. 4c) cells *in vivo*. However, *in situ* imaging and Kaplan-Meyer curves showed a significant reduction in the tumor growth of SVZ-vIII GB cells (Fig. 4d and e). To determine whether the route of administration is relevant for this anti-tumor effect, we tested the compound by intragastric or intravenous administration. The results suggest that using different routes of drug administration did not improve the overall survival of SVZ-vIII treated with VP3.15 (Fig. S1a). Futhermore, we did not find a synergism between VP3.15 and TMZ, the standard GB treatment (Fig. S2).

**Fig. 5:**
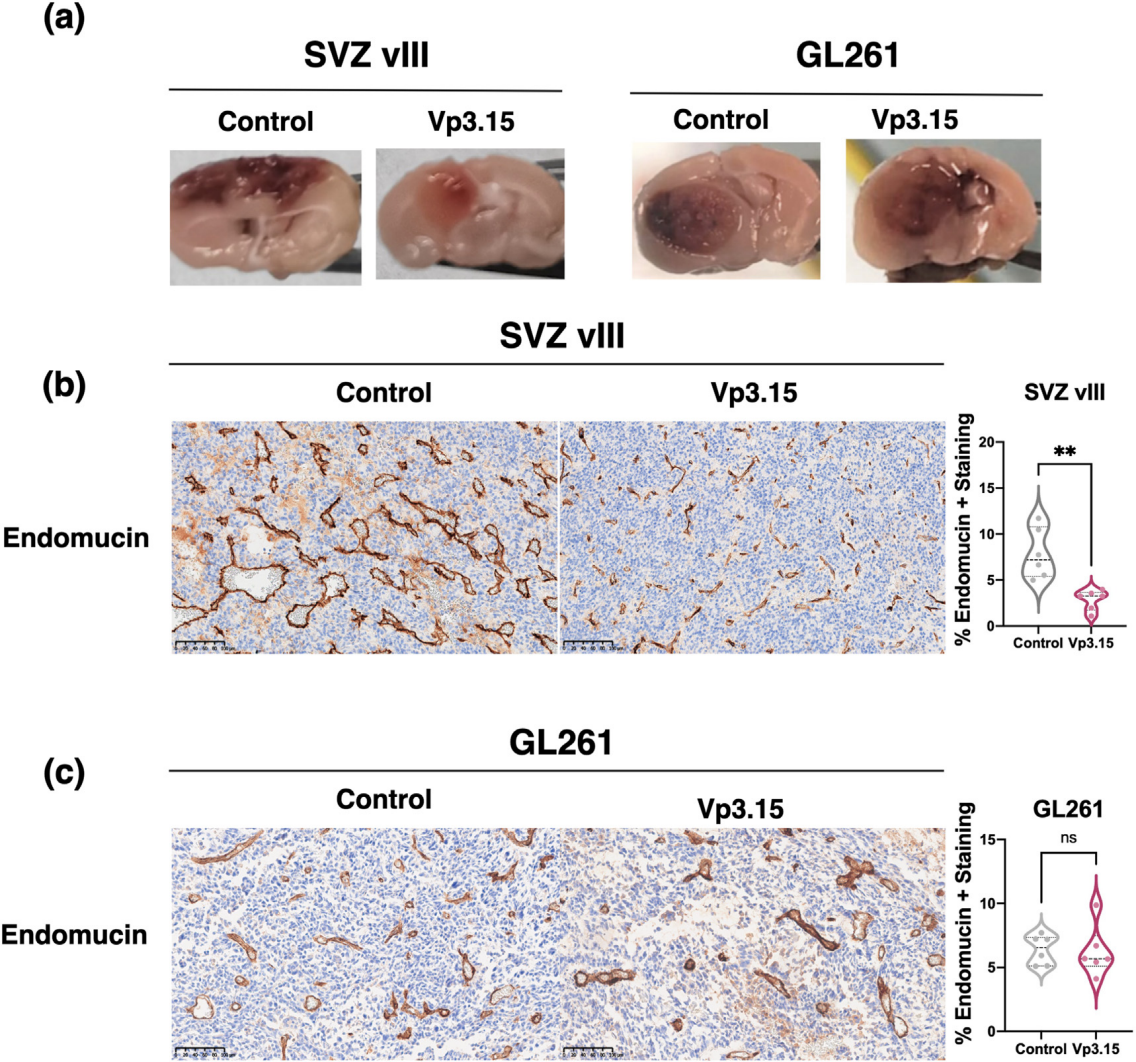
**Vp3.15 effects on the vasculature of SVZ-vIII and GL261 tumors. a)** Representative images of mouse brains carrying SVZ-vIII or GL261 tumors that were treated with the vehicle (Control) or VP3.15. **b-c)** Representative images of endomucin staining of SVZ-vIII **(b)** or GL261 **(c)** tumors. Quantification is shown on the right. ∗∗P ​≤ ​0.01. n.s. non-significant. Scale bar: 100 ​μM.

**Fig S3:**
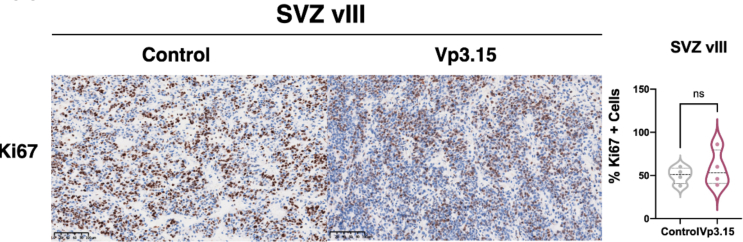
Analysis of the effect of Vp3.15 in the proliferation of SVZ vIII tumors. Representative images of Ki67 staining of SVZ-vIII tumors treated with the vehicle (Control) or VP3.15. Quantification is shown on the right. n.s. non-significant. Scale bar: 100 ​μM

Closed examination of the tumors formed in the mouse brains revealed an apparent reduction in the amount of hemorrhage after VP3.15 treatment in SVZ-vIII, but not in GL261 tumors (Fig. 5a). Consequently, the IHC analysis of the tumor sections confirmed a reduction in the endomucin staining, which marks endothelial cells, in SVZ-vIII (Fig. 5b) but not in GL261 (Fig. 5c) tumors in the presence of the drug. Notably, we did not observe any changes in the number of proliferating cells in SVZ-vIII tumors treated with VP3.15 (Fig. S3).

### The presence of wild-type PTEN is necessary for the anti-tumor effect of VP3.15

**Fig. 6:**
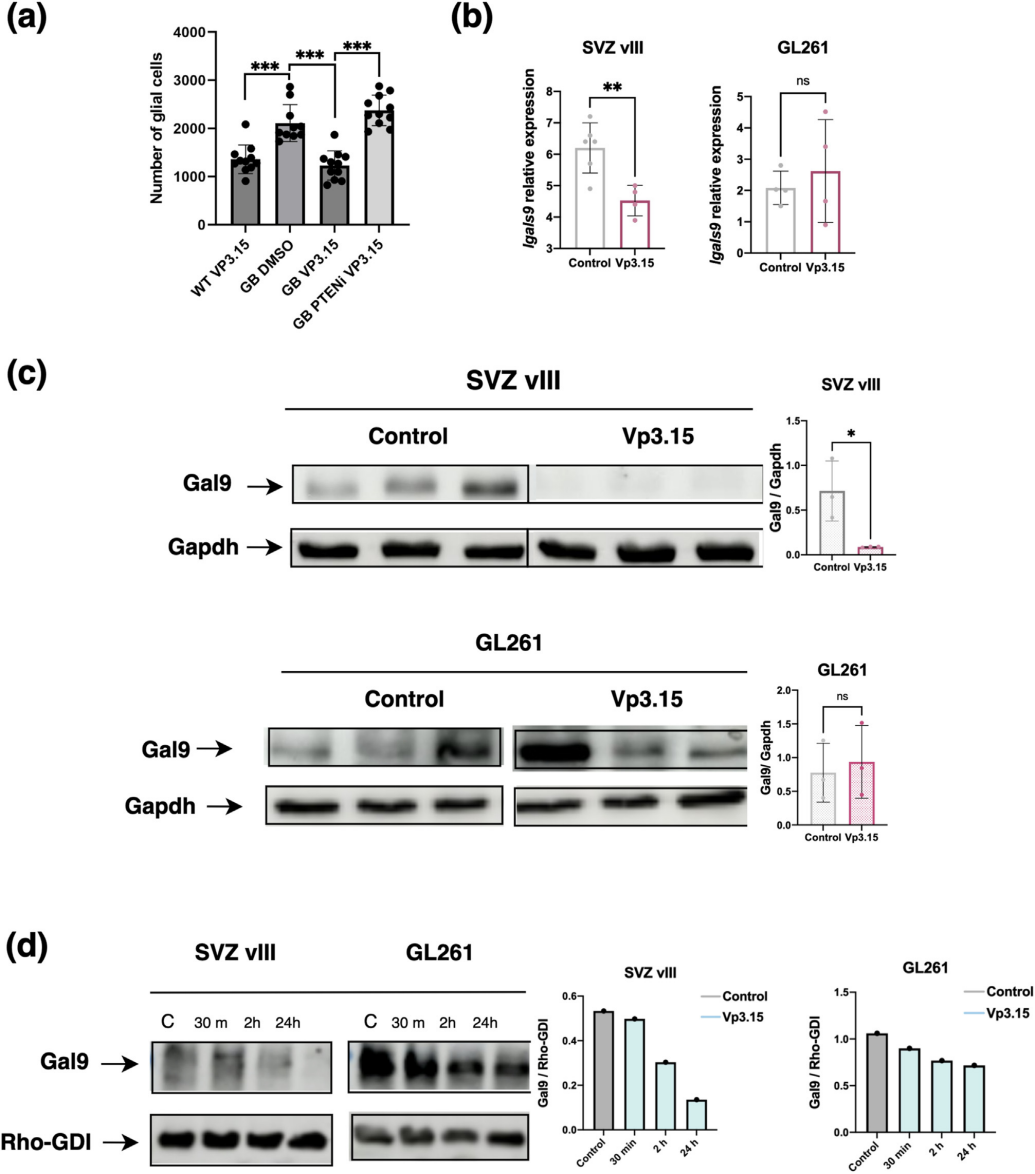
**Analysis of Gal9 expression in response to Vp3.15 treatment in PTEN wild-type and PTEN deficient GB. a)** Number of glial cells in *Drosophila melanogaster* wild type brain samples treated with VP3.15, GB samples, GB samples treated with VP3.15 and GB *Pten*RNAi samples treated with VP3.15. **b)** qRT-PCR analysis of *Lgals9* expression in SVZ-vIII and GL261 tumors treated with vehicle (Control) or VP3.15. *Actin* expression was used for normalization (n ​= ​5). **c)** Western-Blot (WB) analysis to measure the levels of Gal9 in SVZ-vIII and GL261 tumors treated with vehicle (Control) or VP3.15. Gapdh was used as a loading control. Quantification is shown on the right (n ​= ​3). **d)** SVZ-vIII or GL261 ​cells were exposed to 1 ​μM of VP3.15 for 30min, 2 ​h and 24 ​h. Cells were collected and the amount of Gal9 was measured by WB analysis. Rho GDI was used as a loading control. Quantification is shown on the right.

Recent publications suggest that gliomas deficient for the tumor suppressor *PTEN* show a strong inhibition of GSK-3β, which in turn induces the expression of galectin *Gal9* [30]. Notably, the main difference between SVZ-vIII and the other tumors that were not sensitive to VP3.15 is the presence of wild-type *PTEN* (Fig. 3a). To address the relevance of PTEN in the response of GB cells to this inhibitor, we knocked down *Pten* expression in GB cells in the *Drosophila* glioma model using a specific *UAS-Pten RNAi* transgenic line. Fig. 6a shows that the reduction in the number of GB cells in VP3.15 treated flies was reversed in GB ​+ ​*PtenRNAi* flies. Therefore, the results suggest that the anti-GB activity of VP3.15 in *Drosophila* is Pten dependent, although we cannot discard that other factors contribute to the specific anti-tumor response observed in the SVZ-vIII tumors.

In mouse models, we observed a significant downregulation of *Gal9* transcription (Fig. 6b) and protein accumulation (Fig. 6c) in SVZ-vIII tumors treated with VP3.15, but not in GL261 tumors (Fig. 6b and c). To further analyze the effect of the inhibitor on Gal9, we treated GL261 and SVZ-vIII cells in the presence of 1 ​μM of VP3.15 and analyzed the levels of different proteins at different time points. Consistent with published results, the level of *Gal9* expression was significantly higher in the *PTEN*-deficient GL261 ​cells (Fig. 6d). We observed a reduction in the amount of Gal9 protein 24 ​h after the exposure of SVZ-vIII cells to VP3.15. We also observed a similar trend in GL261 ​cells treated with VP3.15, although the remaining levels of Gal9 protein were higher in the PTEN-proficient GB cell line (Fig. 6d).

**Fig. 7:**
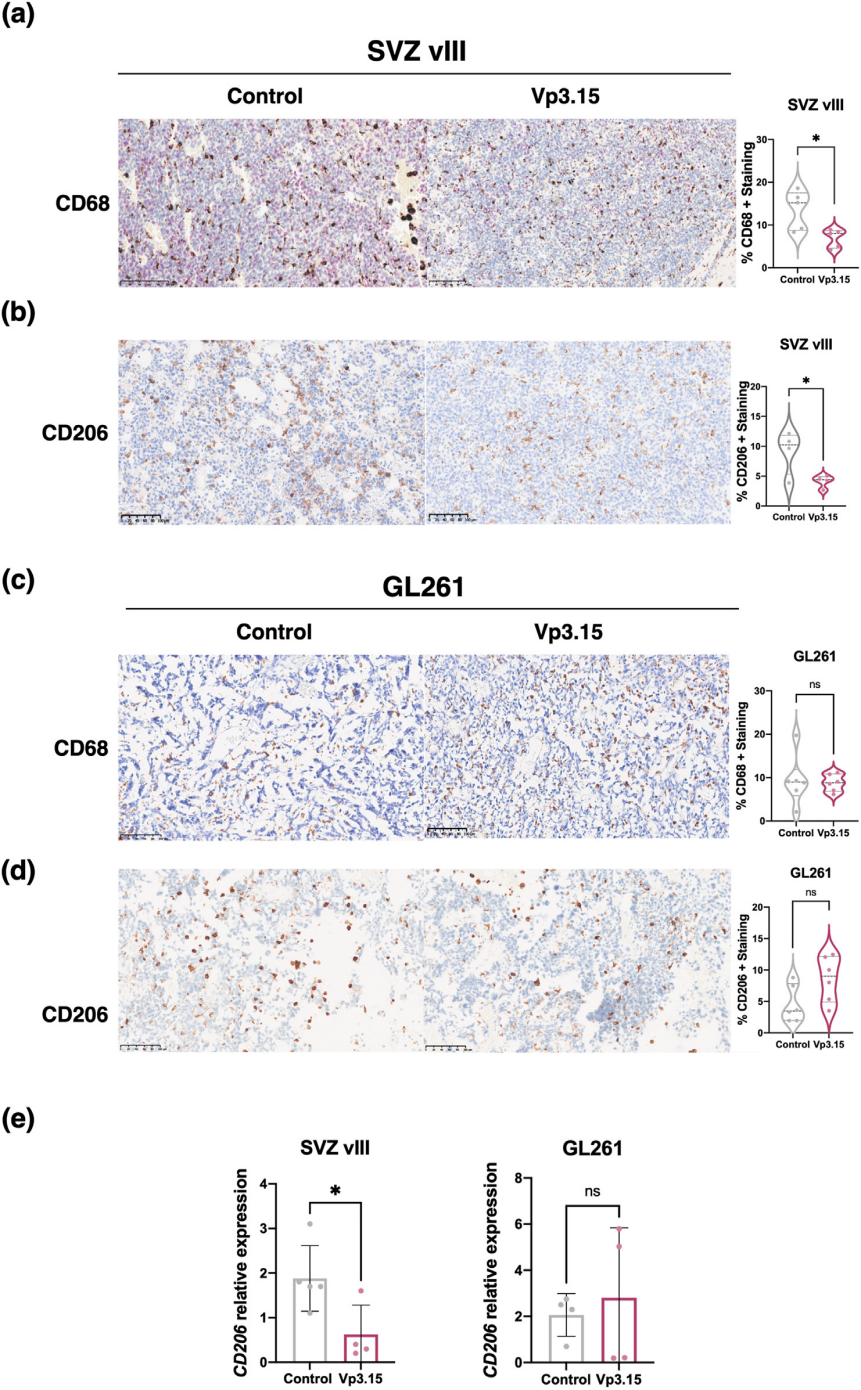
**Analysis of macrophages in response to Vp3.15 treatment in PTEN wild-type and PTEN deficient GB. a-d)** Representative images of CD68 **(a,c)** and CD206 **(b,d)** staining of SVZ-vIII **(a,b)** or GL261 **(c,d)** tumors treated with vehicle (Control) or VP3.15. Quantification is shown on the right. **e)** qRT-PCR analysis of the expression of *CD206* in SVZ-vIII or GL261 tumors treated with vehicle (Control) or VP3.15. Actin was used for normalization. ∗P ​≤ ​0.05. n.s. non-significant. Scale bar: 100 ​μM.

**Fig S4:**
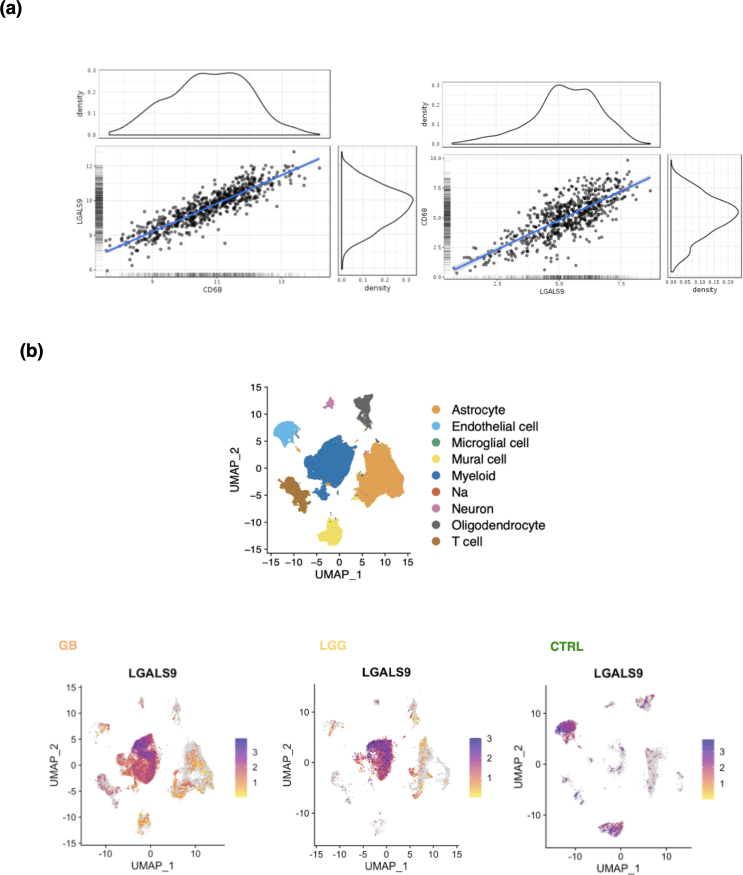
Correlation of *LGALS9* and*CD68* expression in gliomas. a) Data obtained using the GlioVis tools (http://gliovis.bioinfo.cnio.es/) showing the correlation of *LGALS9* and *CD68* expression in the TCGA (The Cancer Genome Atlas) (left) (r ​= ​0.89) and CGGA (Chinese Glioma Genome Atlas) (right) (r ​= ​0.77). Two independent cohorts of glioma samples from patients. b) UMAP plot annotated according to each cell type of the integrated object (control and glioma scRNAseq databases) (top). UMAP plot of cells with positive *LGALS9* expression in normal brain (CTRL), lower-grade gliomas (LGG) and glioblastomas (GB) (bottom).

Galectins are involved in the modulation of cell-cell and cell-matrix interactions. Specifically, secreted Gal9 recruits macrophages that, once inside the tumor tissue, promote GB angiogenesis and growth [30]. Indeed, there is a highly significant correlation between the expression of *LGALS9* (the gene encoding Gal9) and *CD68* (a classical marker of GB macrophages) expression in patient samples from two distinct glioma cohorts (Fig. S4a). Moreover, the scRNAseq integrated atlas (Fig. 1a) was employed to elucidate the pattern of expression of *LGALS9*. Our findings revealed that in the tumors, this gene is expressed extensively, particularly in myeloid cells, yet also to a lesser extent in glial cells. This suggests that multiple cell types may be capable secreting this molecule (Fig. S4b). These observations led us to hypothesize that VP3.15 may influence the population of macrophages within the tumors. To explore this possibility, we quantified the number of myeloid cells through IHC in mouse xenograft GB sections. The results show a significant reduction in the percentage of CD68 (Fig. 7a) and CD206 (Fig. 7b) (a marker of M2 protumoral macrophages [31]) positive cells, in SVZ-vIII tumors treated with VP3.15, but not in GL261 gliomas (Fig. 7c and d). In addition, the expression of *CD206* (measured by qRT-PCR was reduced SVZ-vIII, but not in GL261 tumors treated with the VP3.15 (Fig. 7e). Collectively, these results demonstrate that the anti-tumor effect of VP3.15 in a PTENwt GB is mediated, at least in part, by reducing the recruitment of pro-angiogenic macrophages.

## Discussion

GB is one of the most lethal tumors with a very short life expectancy at diagnosis. Currently, there is no effective treatment, mainly due to its highly heterogeneous and plastic nature [2]. GB is a multifactorial disease and in consequence, combination of different drugs are emerging as promising therapeutic approaches In any case, there is an urgent need to search for new therapeutic strategies, particularly those that could impair the supportive function of the GB microenvironment.

VP3.15 is a small heterocyclic drug-like molecule with safety profile and good pharmacodynamic and pharmacokinetic properties after intraperitoneal administration in different *in vivo* models [32]. Interestingly, it has shown neuroprotection of retinal cells both *ex vivo* and *in vivo* in a model of retinitis pigmentosa [33,34], and neuroprotective and anti-inflammatory properties in models of multiple sclerosis and experimental autoimmune encephalomyelitis [[34], [35], [36]]. Here, we show that VP3.15 inhibits the growth of GB cells *in vitro*, arguing for an anti-proliferative contribution of the drug. Indeed, several groups have shown the cytotoxic effects of GSK-3 blockade, either genetically or with small molecules, in gliomas [[37], [38], [39]]. In spite of the inhibitory action of the PI3K/AKT pathway, commonly hyperactivated in GB, on GSK-3β, most of the previous studies establish a general oncogenic role for GSK-3β in these tumors [37,38]. *GSK-3β* expression is upregulated in GB [40,41], and activating phosphorylation at Tyr216 is present in GB samples [39]. Thus, GB cells show activation of GSK-3β despite the inhibitory effect of GSK-3β^Ser9^ phosphorylation by the PI3K/AKT pathway. This could be explained by the action of additional pathways to prevent GSK-3β^Ser9^ phosphorylation, as it has been previously proposed [42]. Moreover, it was described in chronic neuroinflammation that inhibition of PDE7 by VP3.15, in conjunction with the crosstalk between PDE7 and GSK3, leads to an increase in phosphorylated Ser9 via the PKA pathway. This mechanism suggests that the inhibition of PDE7 can modulate the activity of GSK3 through PKA signaling, promoting Ser9 phosphorylation, which is known to inhibit GSK3 activity [43]. Therefore, the interaction between PDE7 inhibition and the PKA-mediated pathway might play a critical role in regulating GSK3 function through Ser9 phosphorylation.

Apart from its anti-proliferative capacity, our results show that VP3.15 can inhibit the growth of certain gliomas by altering the microenvironment, in particularly by inducing a strong reduction in the number of pro-angiogenic macrophages. A direct effect of VP3.15 on the microglial phenotype has been proposed in autoimmune models [32,44], acting synergistically through GSK-3β and PDE7 inhibition [45]. Given the ubiquitous expression of GSK-3β in gliomas (Fig. 1a), we cannot exclude a direct effect of this molecule on the phenotype of the glioma-associated macrophages. Moreover, it will be interesting to test whether the inhibitor also has a neuroprotective role in gliomas, which could further enhance its chemotherapeutic potential.

Our data suggest that the function of VP3.15 is mediated, at least in part, by reducing the production of Gal9, both in tumors and in glioma cells *in vitro*. Gal9 is a β-galactoside–binding protein involved in the modulation of cell-cell and cell-matrix interactions. Gal9 promotes M2 polarization by activating Tim-3 receptors in macrophages [46]. Recently, Gal9/Tim-3 signaling has been implicated in the glioma-macrophage interactions and, subsequently, in tumor vascularization [30]. Thus, we propose that VP3.15 acts by inhibiting *LGALS9* expression, at least in the presence of wild-type PTEN. Although we cannot discard that other factors could also contribute to the response to the inhibitor, in the *PTEN*-deficient tumors tested here, formed by fly, mouse and human GB cells, we did not observe an anti-tumor effect of the drug. One possible explanation for this could be that PTEN-deficient GB might already have very strong signals to recruit immunosuppressive macrophages. It has been suggested that this could be mediated by LOX regulation [47,48], or even by the inhibition of the GSK-3β-mediated blockade of *Gal9* expression [30]. Therefore, we can hypothesize that GSK-3β might have opposing functions in the regulation of *Gal9* in gliomas, which could depend on the status of PTEN. The findings of our study demonstrate a positive effect on cell viability and in animal models of GB. To date, no evidence of drug resistance has been detected in either the cell cultures or the animal models. Nonetheless, extended longitudinal studies are required to conclusively exclude the possibility of resistance development over time.

The exact role of GSK-3β in malignancies remains highly controversial due to conflicting results frodifferent tumor models. GSK-3β has been shown to function as a tumor suppressor protein that controls cell fate determination and stem cell maintenance by inhibiting the Wnt, Hedgehog, and Notch pathways. These pathways are aberrantly activated in several cancers [[49], [50], [51]]. This implies that GSK-3β inhibitors could potentially exert a therapeutically negative, pro-survival effect on tumor cells. In addition, some studies have found that GSK-3β is part of a tumor suppressor complex consisting of Axin and APC that phosphorylates the oncoprotein β-catenin. If GSK-3β is inactivated, this could potentially lead to tumor promotion [52,53]. Available evidence suggests that GSK-3β may function as a “tumor suppressor” for certain types of tumors such as skin and breast tumors [54,55]. These findings suggest that the mechanisms underlying the function of GSK-3β as a tumor promoter or suppressor may depend on the cell type, tissue context, or, as we propose here, the genetic background of each tumor.

In addition to the inhibiting GSK-3β, VP3.15 acts as an allosteric inhibitor of PDE7. PDE activity is required in GB cells to promote cell proliferation, and therefore the dual inhibitory effect of VP3.15′s may address GB progression through two independent flanks. However, PDE7 inhibition also converges on GSK3β inactivation [43]. Thus, we cannot exclude that the effects of VP3.15 on GB progression are limited to GSK3β inhibition.

In summary, this study highlights a significant pharmacological profile of VP3.15 as a potential disease-modifying agent for gliomas, demonstrating its strong capacity to reduce immunosupportive myeloid infiltration and tumor vascularization. The molecule is orally bioavailable and readily penetrates the blood-brain barrier. These pharmacological properties are particularly encouraging for further development as a drug for adult patients, particularly those with PTENwt gliomas. Nevertheless, the translation of these results into the clinic may open a new avenue to evaluate the efficacy of VP3.15 also in pediatric glioma patients, where Gal9/TIM3 signaling has recently been proven to be a bona fide target [56] and *PTEN* is rarely mutated. A combination with anti-angiogenic molecules that have previously failed to block GB growth [4] may also be worth trying. As this molecule disrupts the supportive tumor microenvironment, tumors are likely to be less susceptible to the emergence of resistant subclones with different genetic alterations.

## Author contribution statement

**Maria Castello-Pons**, Investigation, Methodology, Visualization, Writing-review & editing, **Maria A. Ramirez-Gonzalez**, Investigation, Methodology **Patricia Iglesias-Hernández,** Investigation, Methodology, **Nermina Logo Lendo** Investigation. Methodology, **Carlos Rodriguez-Martín** Investigation, Methodology, **Laura Quiralte** Investigation, Methodology, **Juan-Manuel Sepúlveda-Sánchez,** resources, funding acquisition, **Olaya de Dios**, investigation, methodology, visualization, **Carmen Gil, Ana Martínez** Conceptualization, Methodology, **Pilar Sánchez-Gómez,** Conceptualization, Methodology, Validation, Formal analysis, Investigation, Resources, Writing Original draft, Writing Review & editing, Visualization, Supervision, Project administration, Funding acquisition **and Sergio Casas-Tinto** Conceptualization, Methodology, Validation, Formal analysis, Investigation, Resources, Writing Original draft, Writing Review & editing, Visualization, Supervision, Project administration, Funding acquisition.

## Data availability

All data are available upon request to the corresponding authors.

## Declaration of competing interest

The authors declare no competing interests.
