# Supplementary material for: VP3.15, a dual GSK-3β/PDE7 inhibitor, reduces glioblastoma tumor growth though changes in the tumor microenvironment in a PTEN wild-type context
Source: Neurotherapeutics. 2025 Mar 28;22(4):e00576. doi: 10.1016/j.neurot.2025.e00576 (PMC12418426; doi:10.1016/j.neurot.2025.e00576)
Supplement: Multimedia component 1 [file mmc1.docx]

| Compound name | Chemical structure | GSK-3 IC_50_ (µM) | PDE7 IC_50_ (µM) | Reference |
| --- | --- | --- | --- | --- |
| VP3.15 |  | 0,88 ± 0,07 | 1,59 | (Palomo et al., 2012; Redondo et al., 2012) |
| VP1.14 |  | 1,28 ± 0,35 | 0,38 |  |
| VP1.15 |  | 1,95 ± 0,21 | 1,11 |  |
| S14 |  | __ | 4,68 | (Castaño et al., 2009) |
| TC3.6 |  | __ | 0,55 |  |
| TDZD-8 |  | 2,0 | __ | (Martinez et al., 2002) |
| Tideglusib |  | 0,06 | __ | (Domínguez et al., 2012) |
| VP2.51 |  | 0,62 ± 15 | __ | (de Munck et al., 2016) |
| VP0.7 |  | 2,57 ± 0,19 | __ | (Palomo et al., 2017) |

**S1a. Chemical structure and target inhibition of the compounds used in this study**

**S1b. Key resources table**

| **Antibodies** |  | **SOURCE** | **IDENTIFIER** | **DILUTION** |
| --- | --- | --- | --- | --- |
| **IHC** |  |  |  |  |
| mAnti-Endomucin | | Santa Cruz Biotehechology | sc-65495 | 1:600 |
| mAnti-Ki-67 |  | Cell Signaling | #12202 | 1:600 |
| mAnti-CD206/MRC1 | | Cell Signaling | #91992 | 1:500 |
| mAnti-CD68 |  | Cell Signaling | #97778 | 1:600 |
| **WB** |  |  |  |  |
| mAnti-GAPDH |  | Santa Cruz Biotehechology | sc-47724 | 1:1000 |
| mAnti-Rho |  | Santa Cruz Biotehechology | sc-313724 | 1:1000 |
| mAnti-galectin 9/Gal-9 | | Abcam | ab275877 | 1:1000 |
| mAnti-mouse IgG, HRP-linked | | Cell Signaling | #7076 | 1:2000 |
| mAnti-rabbit IgG, HRP-linked | | Cell Signaling | #7074 | 1:2000 |
|  |  |  |  |  |
| **Commercial assays** | | **SOURCE** | **IDENTIFIER** |  |
| ReliaPrep™ RNA Cell Miniprep System | | Promega | Z6010 |  |
| ReliaPrep™ RNA Tissue Miniprep System | | Promega | Z6110 |  |
| PrimeScript™ RT Reagent Kit | | Takara | RR037B |  |
| TB Green® Premix Ex Taq™ II | | Takara | RR82WR |  |
| BCA Pierce |  | Thermo Scientific | #23225 |  |
| BOND RXm |  | Leica Biosystems | BOND Instrument Consumables | |
| **Oligonucleotides** | | **SOURCE** |  |  |
|  |  | Sigma-Aldrich |  |  |
|  |  |  |  |  |
| **Gene** | **Fwd Primer** |  | **Rev Primer** |  |
| **House-Keeping** |  |  |  |  |
| GAPDH | GTCTCCTCTGACTTCAACAGCG | | ACCACCCTGTTGCTGTAGCCAA | |
| **Interest Genes** |  |  |  |  |
| lGals9 | CTGGAATCCCTCCTGTGGTGTA | | CCTCGTAGCATCTGGCAAGACA | |
| CD206/MRC1 | AGCCAACACCAGCTCCTCAAGA | | CAAAACGCTCGCGCATTGTCCA | |
|  |  | |  | |

**S1c. Leica Bond DAB protocol**

| **Immunohistochemistry (Leica® BOND™)** | | |  |
| --- | --- | --- | --- |
| **NOTE**: Please see product datasheet or product webpage for appropriate antibody dilution^. | | | |
|  |  |  |  |
|  | **Step** | **Reagents** | **Time/Temperature** |
| 1 | Dewax | BOND™ Dewax Solution, 100% Alcohol, BOND™ Wash Solution | Pre-programmed Leica® BOND™ |
| 2 | Antigen Retrieval | BOND™ Epitope Retrieval ER2 Solution | 20 min., 100˚C \| Protocol: HIER 20 min with ER2 |
| 3 | Peroxide Block | Refine Detection Kit Peroxide Block* | 5 min. |
|  | WASH | BOND™ Wash Solution | 3x 0:00 min. |
| 4 | Protein Block (optional) | #5425 NGS or #15019 Animal-Free Blocking Solution | 20 min. |
| 5 | Primary Antibody^ | Dilute in #8112 SignalStain® Antibody Diluent | 30 min. |
|  | WASH | BOND™ Wash Solution | 3x 2:00 min. |
| 6 | Secondary Detection | Refine Detection Kit Polymer* | 10 min. |
|  | WASH | BOND™ Wash Solution/Deionized Water | Custom (see below) |
| 7a | Visualization | Refine Detection Kit Mixed DAB Refine* | 0:00 min. |
| 7b | Visualization | Refine Detection Kit Mixed DAB Refine* | 10 min. |
|  | WASH | Deionized Water | 3x 0:00 min. |
| 8 | Counterstain | Refine Detection Kit Hematoxylin* | 5 min. |
|  | WASH | Deionized Water | 0:00 min. |
|  | WASH | BOND™ Wash Solution | 0:00 min. |
|  | WASH | Deionized Water | 0:00 min. |
| 9 | Dehydration (Offline): |  |  |
|  | Incubate sections in 95% ethanol two times for 10 seconds each. | | |
|  | Repeat in 100% ethanol, incubating sections two times for 10 seconds each. | | |
|  | Repeat in xylene, incubating sections two times for 10 seconds each. | | |
| 10 | Mount sections with coverslips and #14177 SignalStain® Mounting Medium | | |
|  |  |  |  |
|  | Optional Custom wash: | BOND™ Wash Solution | 2:00 |
|  |  | BOND™ Wash Solution | Dispenser Type: OPEN 0:00 |
|  |  | BOND™ Wash Solution | 2:00 |
|  |  | BOND™ Wash Solution | Dispenser Type: OPEN 0:00 |
|  |  | BOND™ Wash Solution | 0:00 |
|  |  | Deionized Water | 0:00 |
| *Reagent included in BOND™ Polymer Refine Detection Kit (Catalog No: DS9800) | | | |
| LEICA® is a registered trademark of Leica Microsystems IR GmbH. | | | |
| BOND™ is a trademark of Leica Biosystems Melbourne Pty. Ltd. No affiliation or sponsorship between CST and Leica Microsystems IR GmbH or Leica Biosystems Melbourne Pty. Ltd is implied. | | | |
| posted August 2018 | | | |
| revised September 2018 | | | |
| Protocol Id: 1444 | | | |
|  |  |  |  |

**S1d. QuPath quantification settings**

| **Positive-cell detection** |
| --- |
| Create positive cell detection with the following settings: |
| **Endomucin** |
| Detection image: Optical density sum |
| Requested pixel size: 0.5 μm |
| Background radius: 10 μm |
| Median filter radius: 2 μm |
| Sigma: 2 μm |
| Minimum area: 10 μm^2 |
| Maximum area: 400 μm^2 |
| Threshold: 0.2 |
| Max background intensity: 2 |
| Cell expansion: 2μm |
|  |
| **Pixel classification method** |
| Create thresholder with the following settings: |
| **CD68** |
| Resolution: Full (0,22μm/px) |
| Channel: DAB |
| Prefilter: Gaussian |
| *Smoothing* sigma: 2 |
| *Threshold*: 0.27 |
| Region: Any object ROI |
| Above *Threshold*: Positive |
| Below *Threshold*: Ignore* |
| **CD206** |
| Resolution: Full (0,22μm/px) |
| Channel: DAB |
| Prefilter: Gaussian |
| *Smoothing* sigma: 2 |
| *Threshold*: 0.27 |
| Region: Any object ROI |
| Above *Threshold*: Positive |
| Below *Threshold*: Ignore* |
